# Characterization of Autoimmune Thyroid Disease in a Cohort of 73 Paediatric Patients Affected by 22q11.2 Deletion Syndrome: Longitudinal Single-Centre Study

**DOI:** 10.3390/genes13091552

**Published:** 2022-08-28

**Authors:** Silvia Ricci, Walter Maria Sarli, Lorenzo Lodi, Clementina Canessa, Francesca Lippi, Chiara Azzari, Stefano Stagi

**Affiliations:** 1 Department of Health Sciences, University of Florence, Paediatric Immunology Division, Anna Meyer Children’s University Hospital, 50139 Florence, Italy; 2 Paediatric Immunology Division, Anna Meyer Children’s University Hospital, 50139 Florence, Italy; 3 Department of Health Sciences, University of Florence, Auxoendocrinology Division, Anna Meyer Children’s University Hospital, 50139 Florence, Italy

**Keywords:** 22q11.2 deletion syndrome, Di George syndrome, hypothyroidism, hyperthyroidism, microdeletion, pediatric, thyroid disease, autoimmune thyroid disease, thyroid sonography, thyroid hypoplasia

## Abstract

Background. Chromosome 22q11.2 Deletion Syndrome (22q11.2DS) is the most frequent microdeletion syndrome and is mainly characterized by congenital cardiac defects, dysmorphic features, hypocalcemia, palatal dysfunction, developmental delay, and impaired immune function due to thymic hypoplasia or aplasia. Thyroid anomalies are frequently reported in patients with 22q11.2DS, although only a few well-structured longitudinal studies about autoimmune thyroid disease (ATD) have been reported. Aim. To longitudinally evaluate the frequency of thyroid anomalies and ATD in patients with 22q11.2DS. Patients and Methods. Pediatric patients with a confirmed genetic diagnosis of 22q11.2DS were recruited and followed up on longitudinally. Clinical, biochemical, and immunological data were collected, as well as thyroid function, autoimmunity, and thyroid sonographic data. Results. The study included 73 children with 22q11.2DS, with a mean follow-up duration of 9.51 ± 5.72 years. In all, 16 of the 73 enrolled patients (21.9%) developed ATD before 18 years of age (mean age 12.92 ± 3.66 years). A total of 20.5% developed Hashimoto’s Thyroiditis (HT), of whom 50% required L-thyroxine treatment; 1.4% developed Graves Disease. Thyroid hypoplasia was found in 6/16 patients with ATD and left lobe hypoplasia in 9/16 patients. These features were also found in patients affected by 22q11.2DS without ATD. Among patients who developed ATD, at the first altered ultrasound scan, the most frequent anomalies suggestive of thyroiditis were inhomogeneous echotexture, diffuse or irregular hypo-echogenicity, and vascular overflow. Conclusion. We strongly recommend periodic screening of thyroid function and for autoimmunity in patients affected by 22q11.2DS. Along with blood tests, ultrasound scans of the thyroid gland should be performed periodically since some patients who go on to develop an ATD could have specific anomalies on ultrasound prior to any other anomaly.

## 1. Introduction

Chromosome 22q11.2 Deletion Syndrome (22q11.2DS) is the most frequent microdeletion syndrome, with an incidence rate ranging from 1:4000 to 1:10,000 live births worldwide [1,2]. It is caused by a small hemizygous deletion, which is de novo in more than 90% of cases [1].

22q11.2DS embraces a group of phenotypically similar diseases characterized by abnormal development of the third and fourth branchial arches, resulting in variable combinations of congenital cardiac defects, dysmorphic features, hypocalcemia, palatal dysfunction, developmental delay, neuropsychiatric disorders, and impaired immune function due to thymic hypoplasia or aplasia [3,4].

Thyroid anomalies are also frequent in patients with 22q11.2DS. Approximately half of patients have structural anomalies such as thyroid hypoplasia, absent isthmus, and abnormal extension, probably due to *TBX1* (OMIM * 602054), which is mapped on 22q11 haploinsufficiency [5,6,7]. Thyroid dysfunction can also be caused by autoimmunity. The gland can be directly infiltrated by self-reactive lymphocytes or damaged by self-reactive autoantibodies against thyroid peroxidase (TPOAb), thyroglobulin (TGAb), or TSH receptor (TRAb). It is estimated that over 8% of patients with 22q11.2DS will develop autoimmunity with age [8].

The thymus is fundamental for T lymphocyte proliferation and development as within this organ, positive and negative selection of T cells takes place. It is known that inside the thymus, the autoimmune regulatory gene (*AIRE1*; OMIM * 607358) upregulates the expression of organ-specific self-antigens by thymic epithelial cells to achieve central tolerance toward these self-antigens [9]. Since some lymphocytes with intermediate affinity to self-antigens could escape from the thymus, especially if it is hypoplastic, it is important to have peripheral mechanisms of tolerance. Peripheral tolerance is obtained by ignorance, deletion, anergy, or regulation. Autoimmune diseases could also be attributable to a reduction in thymic-derived CD4+CD25+T regulatory lymphocytes [10].

For these reasons, patients affected by 22q11.2DS have a higher rate of thyroid and non-thyroid autoimmune diseases in addition to a higher rate of infections. Autoimmune thyroid diseases (ATD) are the most common autoimmune disorders affecting 2–5% of the general population, with greater predominance in women (5–15%) than in men (1–5%) [11,12]. There is also a familiar predominance [13]. Graves’ Disease (GD) is rarer than Hashimoto’s Thyroiditis (HT), particularly before puberty. According to Shugar et al. [14], in children with 22q11.2DS, there is a higher rate of hypothyroidism (7.7%) and hyperthyroidism (1.8%) than in the general population. Most ATD in pediatric patients with 22q11.2DS is subclinical, but since thyroid autoantibody production increases over time, a significant number of those with subclinical thyroid disease will progress to an overt disease requiring therapy.

## 2. Materials and Methods

This is an observational, prospective, longitudinal, single-center study. The study recruited patients with a confirmed genetic diagnosis of 22q11.2DS, aged under 18 years at diagnosis, from the Paediatric Immunology Division and Endocrinology Division, Meyer Children’s Hospital in Florence, Italy, between 1 April 1985 and 7 June 2022. All patients were monitored annually or biannually up to the age of 18 through clinical assessments, abdominal or thyroid ultrasounds, and blood tests to measure thyroid function and autoantibodies.

Clinical, biochemical, and immunological data (antinuclear antibodies and lymphocyte subsets) were collected from clinical records for all patients during follow-up. The lymphocyte-specific population was defined as follows. T cells: CD4+CD45RA+ naïve helper T cells, CD4+CD45RO+ activated/memory helper T cells, CD4+CD45RA+CD31+ Recent Thymic Emigrants (RTEs) (expressed as percentage of naïve CD4+ T cells). B cells: CD27+IgM-IgD- switched memory B cells (SMB) and total CD19+ B cells.

Written, informed consent from the patients’ parents or legal guardians was acquired before data collection. Specific approval by the local ethical committee was not required because all analyses included in this study were performed as part of routine clinical activity, according to Good Clinical Practice.

### 2.1. Disease Definition

Diagnosis of ATD, in agreement with the literature [15,16], was established by two or more of the following clinical/laboratory signs:

(a) Positive serum thyroid autoantibodies against TPOAb, TGAb, and/or TRAb;
(b) Characteristic ultrasound scan with inhomogeneous echotexture, diffuse or irregular hypo-echogenicity (typical for thyroiditis);
(c) Hormonal alteration on blood essays or clinical manifestations suggestive of hormonal alteration.

All blood samples were sent to our laboratory to ensure that all samples were analyzed with the same methods and with the same laboratory equipment. FT_4_, FT_3_, and TSH serum levels were determined by immunometric assays. Overt hypothyroidism was defined as a persistently increased TSH with a decreased serum thyroid hormone level. Thyroid autoimmunity was evaluated by fluorescence enzymatic immunoassays of TG and TPO antibodies; TRAb was measured with a two-step radioreceptor assay [7].

All ultrasound scans were performed in our hospital. Thyroid volume was determined using the ellipsoid volume formula: Pi/6 × length × width × depth, separately for each lobe. Total thyroid volume was calculated as the sum of the right lobe volume and the left lobe volume. The volume of the isthmus was not included. Thyroid hypoplasia was defined as −2 SD of the normal thyroid volume [7]. Color flow ultrasonography was performed on each patient to estimate thyroid vascularization. Follow-up was interrupted in a small number of patients before they were 18 due to familial or socio-economic issues; thus, only 42/57 ultrasound scans were available for patients who did not develop ATD.

Drug use that could possibly influence thyroid function was investigated and excluded.

For each enrolled patient demographic features, age of onset of ATD, quantitative values of thyroid autoantibodies, thyrotropin, features of first altered thyroid ultrasound, therapy, non-thyroid features such as antinuclear antibodies, immunophenotyping, characteristics of cardiopathy, and other autoimmunity manifestations, were collected and analyzed.

### 2.2. Statistical Analyses

We used SPSS ver. 25.0 (SPSS Inc. Chicago, IL, USA) for statistical analysis. A descriptive statistical analysis was performed to summarize the variables of interest. Quantitative variables are expressed as mean and standard deviations (DS) or median and interquartile range (IQ). The results were compared with Student’s *t* test. Fisher test and χ^2^ were used to evaluate differences between the groups when appropriate. The level of significance was set to *p* < 0.05.

## 3. Results

The study included 73 children (0–18 years) affected by 22q11.2DS (genetic diagnosis made with FISH 29/73, CGH array 27/73, FISH/CGH array 10/73, for 7/73, no data for genetic analyses were available).

The study is based on a long follow-up period (694 patient-years); the mean duration of follow-up for enrolled patients was 9.51 ± 5.72 years, the minimum follow-up period was 1 year, and the maximum follow-up period was 18 years. The mean age at diagnosis of 22q11.2DS was 2.27 years with a median age of 0 months (IQ 0–36) due to neonatal cardiac involvement, which was the first onset sign in 52% of patients (Table 1). In all, 16 of the 73 enrolled patients (21.9%) developed ATD before 18 years of age (15/16 20.5% HT and 1/16 1.4% GD) (Table 2). This percentage would have increased to 27% if we had included the four patients who showed some initial isolated alteration on an ultrasound scan or isolated elevation in thyrotropin during a follow-up but did not have autoantibodies or overt ATD requiring therapy until shortly after they turned 18. In total, 9 out of 16 patients required therapy, with a mean age of 12.92 ± 3.66 years.

### 3.1. Thyroid Ultrasound Scan

**Figure 1:**
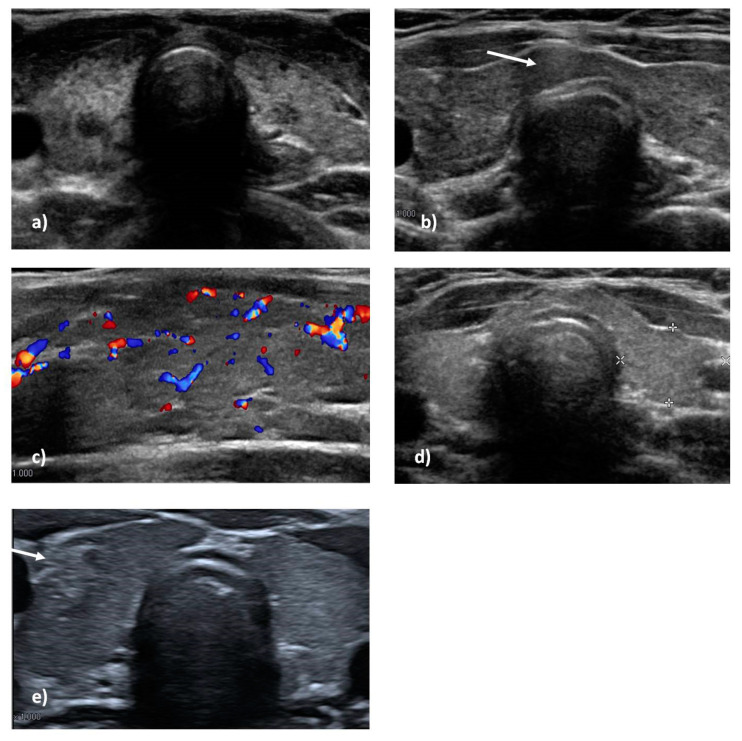
Cervical ultrasonography in some patients with 22q11.2 Deletion Syndrome: thyroid gland hypoplasia as well as inhomogeneity, diffuse hypoechoic areas, and pseudonodular pattern (**a**); diffuse inhomogeneity, hypoechogenity, and perithyroidal lymph node (white arrow) (**b**). Increased overflow of thyroid lobe on color doppler ultrasonography (**c**). Left lobe hypoplasia (**d**); right thyroid lobe neoplasia in a patient with Graves’ disease (white arrow) (**e**).

For each patient, thyroid structural features were collected, and signs compatible with ATD were looked for. Tridimensional measures of thyroid lobes were used to calculate the thyroid lobes’ volume and the total volume. Values were normalized for body surface and height [17,18]. As mentioned, we found thyroid hypoplasia in 6/16 patients with ATD; the left lobe was smaller than the right lobe in 9/16 patients (mean value −0.57 mL); left lobe hypoplasia persisted in 8/16 patients in further ultrasound scan controls (Figure 1). These features were also found in patients affected by 22q11.2DS without ATD. No statistical differences were found between the two groups. However, morphologic anomalies, such as hypoplastic isthmus or lumpy appearance, were described more frequently in patients with progressive ATD (7/16 v. 7/42); this difference was statistically significant (*p* 0.0096). The only patient with GD developed thyroid cancer a few months after diagnosis and shortly after starting adequate therapy.

Among patients who developed ATD, at the first altered ultrasound scan, the most frequent anomalies suggestive of thyroiditis were inhomogeneous echotexture (13/16), diffuse or irregular hypo-echogenicity (10/16), and vascular overflow (9/16) (Figure 1 and Table 3). Four patients had a pseudo-nodular pattern, and two of these had a hypo-echoic nodule. In all, 12/16 patients also had reactive submandibular or cervical lymphadenopathies; interestingly, 4/16 patients had peri-thyroidal lymphadenopathies. After a first altered ultrasound, and even after beginning therapy, 12/16 patients were subjected to further ultrasound scans, and the anomalies mentioned above persisted over time: inhomogeneous echotexture (11/12), diffuse or irregular hypo-echogenicity (10/12), and vascular overflow (8/12). Reactive parathyroid lymphadenopathies persisted in 4/12 patients, in 3 of whom they persisted even after 3–6 years of therapy (Figure 1 and Table 3).

Among patients who did not develop ATD and who had an ultrasound during follow-up, the most frequent anomalies were inhomogeneous echotexture (14/42) and diffuse or irregular hypo-echogenicity (8/42). Nodules were found in 4/42 patients. Although rare, overflow was described in some patients (2/42), as were parathyroid lymphadenopathies (2/42) (Figure 1 and Table 3). The ultrasound differences between the two groups of patients are shown in Table 3.

### 3.2. Thyroid Function and Autoantibodies

The mean values of TG and TPO autoantibodies among patients who developed HT were 360.42 ± 254.06 and 302.49 ± 303.20, respectively. The highest mean values of TSH were 13.03 ± 18.09, and the lowest values of FT_4_ were 0.94 ± 0.20. The only patient who developed GD had a maximum TRAb autoantibodies value of 9.47 with undetectable thyrotrophin at diagnosis. In all, 4/5 patients with elevated TPO autoantibodies (>100) and elevated TSH (>8) at diagnosis progressed in a few months to require therapy.

### 3.3. Risk Factors Analysis for ATD in the 22q11.2 Pediatric Population

The female/male ratio was 13/16 in ATD 22q11.2 patients and 23/57 in non-ATD patients. The fisher exact test showed a *p*-value of 0.004.

Among the 15 patients who developed HT, the mean age at the appearance of TGAb was 9.73 ± 4.29 years, and the mean age at the appearance of TPOAb was 11.57 ± 3.81 years. A total of 67% of these patients were positive for both TPOAb and TGAb, while 20% and 13% had only TGAb or TPOAb, respectively. The mean age at the first elevation of TSH was 9.44 ± 2.80 years among patients who developed HT. The only patient who developed GD showed positive antibodies against the TSH receptor (TRAb) and a reduction in TSH at 11.3 years. The mean age at the first altered ultrasound scan was 10.98 ± 2.97 years.

The mean age of onset of ATD was 10.34 ± 2.87 years. We noticed that most patients who developed ATD had positive ANA with a titer higher than 1:160 (12/16 in ATD 22q11.2DS patients against 23/57 in non-ATD patients). The fisher exact test showed a *p*-value of 0.003.

No statistical difference was found for associations with other autoimmunity conditions between the two groups. The most frequent association was with dermatological autoimmunity (alopecia, psoriasis, vitiligo), followed by coeliac disease, juvenile idiopathic arthritis, and Evans syndrome.

In all, 46/73 patients had a cardiac defect, mostly diagnosed at birth. We analyzed all the data and found that most patients had a combined defect (25/46). The most frequent congenital heart diseases were IVD or Fallot Tetralogy. Overall, 5/16 patients who developed ATD had severe pluri-operated cardiopathies compared to 27/57 patients who did not develop autoimmunity. No statistical differences were found between the two groups.

We considered the lowest lymphocyte count for each patient to establish if lymphopenia could be a risk factor for developing thyroid autoimmunity. We also analyzed lymphocyte subsets through flow cytometry and considered the nadir value of each subset. A total of 28.7% of patients with 22q11.2 showed lymphopenia, mostly in the CD3+ or CD3CD4+ subsets. Among patients who developed ATD, 6/16 had lymphopenia compared to 15/57 of patients who did not develop ATD. The Student’s *t* test found no difference between the two groups of patients for the CD3+ or CD4+ subsets. A statistical difference was found in CD19+ levels, which were lower in the group of patients who developed ATD. Naïve CD4 T-cells known as Recent Thymic Emigrants (RTE CD3 + CD4 + CD45RA + CD31+) as well as CD27 + IgM + IgD- switched memory B cells were analyzed, but no statistical differences were found either in the number of RTE or in switched memory B cells between the two groups of patients. Mean values with DS and *p*-values for each subset are expressed in Table 4.

## 4. Discussion

In agreement with the literature, we found that patients affected by 22q11.2DS frequently have thyroid dysmorphisms [7]. Morphologic structural features of the gland have frequently been described in association with this syndrome, and, as stated in our previous studies [7], these could be linked to the syndrome and not to a specific mechanism of autoimmunity against the thyroid [7]. A study conducted with a multiplanar computed tomography scan suggested the presence of thyroid dysmorphisms in 22q11.2 DS patients, with a frequency of nearly 50% of total cases [6]. Small glandular volume and left lobe hypoplasia were the most frequent morphological and structural features, but we found that patients who developed ATD had morphological anomalies such as a small isthmus in ultrasound scans more often than those that did not develop ATD. The most precocious ultrasound anomalies suggestive of thyroiditis were inhomogeneous echotexture and diffuse or irregular hypo-echogenicity. These features were also found in patients who did not develop ATD, showing low specificity. However, the presence of overflow, as well as parathyroid lymphadenopathy, was more frequently described in patients who developed ATD with statistical significance. Indeed, parathyroid lymphadenopathy is described as being associated with inflammatory thyroid diseases and is rarely detected in other thyroid diseases. For this reason, it is a good indicator of thyroiditis [19]. Overflow or parathyroid lymphadenopathy on ultrasound strongly suggests ATD and should lead to a prompt follow-up to avoid consequences for growth and development, even in the absence of positive serology or clinical signs or elevation/reduction in thyrotropin. Occasionally, an altered thyroid ultrasound has been described prior to the elevation of thyroid autoantibodies or thyrotropin in the general population [20,21,22,23,24]. Many of our patients who developed ATD over time had a history of initial isolated ultrasound anomalies casually found during follow-up. In all, 5/16 patients who developed ATD showed hormonal alteration such as thyrotropin elevation after the appearance of positive autoantibodies or anomalies on an ultrasound scan. Thus, follow-up should not be based only on periodic evaluation of TSH or FT_4_ but also on periodic ultrasound scans and serology. It is possible, moreover, in patients with 22q11.2DS and predisposed to autoimmunity, that inflammation of unknown etiology unmasks the thyroid antigens and leads to the production of autoantibodies and then to overt ATD.

Driscoll et al. [25] and Wilson [26] noted hypothyroidism in 1/15 (7%) and 2/44 (5%) patients with 22q11.2DS, respectively, while a large European collaborative study found hypothyroidism in only 4/548 (0.7%) patients with 22q11.2DS [27]. According to Shugar et al. [14] children with 22q11.2DS have a higher rate of hypothyroidism (7,7%) and hyperthyroidism (1,8%) than the general population. In this study, we noticed that 21.9% of patients affected by 22q11.2DS developed ATD before 18 years of age (15/16 HT and 1/16 GD). This percentage would have been 27% if we had prolonged follow-up since four patients, who had shown some initial isolated alteration on ultrasound or isolated elevation in thyrotropin during our follow-up, had autoantibodies and developed overt ATD requiring therapy soon after turning 18 (Table 5). They were not considered in the group of ATD patients since diagnosis was made after 18 years.

This significantly higher prevalence in our study population could be explained by the prolonged follow-up period, which included frequent blood thyroid assessment and ultrasound scans even without evident clinical signs of thyroid disease. In the general population, most cases of HT are diagnosed in women between 30 and 50 years, with a peak incidence in men occurring 10–15 years later. A similar peak age of onset is described for GD. The mean age of onset of ATD in our cohort was 10.34 ± 2.87 years, in keeping with the age of onset described in the literature for patients with 22q11.2DS. The significantly high incidence of early onset ATD among patients affected by 22q11.2DS could be attributable to the high predisposition for autoimmunity in these patients and/or the specific follow-up they were subjected to. As mentioned, all patients with a diagnosis of 22q11.2DS were evaluated frequently during the follow-up. For this reason, ATD was recognized early, generally before clear clinical signs of thyroidopathy.

Different variables of interest were analyzed as possible risk factors, and statistical significance was found only for two of them: female sex and antinuclear antibodies with a titer higher than 1:160. As expected, in agreement with data for the general population, there was an important female sex predominance, and female sex was one of the strongest risk factors for developing ATD. Antinuclear positivity, especially with a high titer, was the second strongest risk factor.

Severe pluri-operated congenital heart defects could determine a further thymic dysfunction in patients with partial 22q11.2DS due to thymectomy and increase their risk of autoimmunity. In a previous study [7] we suggested a possible association between thyroid disease and cardiac anomalies in patients with 22q11.2DS, but here, we found no statistical correlation between ATD and severe pluri-operated cardiac disease, in agreement with Shugar et al. [14]. Furthermore, we found no correlation with T-cell lymphopenia, anomalous values of RTE, or switched memory B cells. Although most lymphopenic patients with 22q11.2DS do not develop autoimmune thyroid disease, we know that lymphopenia could determine an imbalance in T-cell homeostasis, which can contribute to a breaking of self-tolerance [19,29,30,31,32]. As postulated in our previous study [33] and by Tison et al. [34] and Montin et al. [35] a low number of RTE or anomalous values of memory B cells could also be predictive of a higher risk of autoimmunity. We noted that patients who developed ATD had lower values of CD19+ B lymphocytes. Stozek et al. demonstrated that a reduction in the number of B-regs with CD19 + CD24 + CD27 + IL-10+ and CD19 + IL-10+ expression could break immune tolerance and lead to ATD development in children [36]. We did not analyze this specific subset of B lymphocytes, but it is reasonable to suppose that a reduction in total B cell values could also determine a reduction in B-regs.

To the best of our knowledge, this is the first study of selective characterization of ATD in patients with 22q11.2DS that focused on immunophenotyping. Even though it is only a hypothesis, ATD could be different from all other autoimmune diseases previously studied in patients with 22q11.2DS and depend on pathologic mechanisms not yet understood. In the general population, there is a high frequency of ATD associated with systemic autoimmune disorders such as systemic lupus erythematosus (SLE) or other organ-specific autoimmunity disorders, such as type 1 diabetes or coeliac disease. The association of autoimmune diseases in the same patient or family could be explained by a shared genetic HLA predisposition or single nucleotide polymorphisms of various genes involved in immune regulation [37]. In our patients with ATD, we did not find any relevant association with other autoimmune diseases. The reasons for this difference between patients with 22q11.2DS and the general population are not completely known. It is probable, as mentioned above, that autoimmunity in 22q11.2DS is multifactorial and requires a combination of different breaks in self-tolerance. Therefore, ATD in a patient with this syndrome could combine over time with any other autoimmune disease without preferential associations or presentation patterns. Patients should be closely followed and screened periodically even after turning 18 to permit early diagnosis and treatment for autoimmunity.

## 5. Conclusions

This study emphasizes the importance for patients affected by 22q11.2DS of endocrine evaluation at diagnosis and during follow-up to diagnose ATD early and to ensure proper and precocious treatment. As for many other syndromes such as Noonan, Down, Williams, Turner, and Klinefelter, a complete follow-up is strongly recommended for patients affected by 22q11.2DS, including blood tests with thyroid function (TSH, FT_4_, FT_3_) and autoimmunity (TGAb, TPOAb, TRAb autoantibodies) annually or biannually. Along with blood tests, ultrasound scans of the thyroid gland should be performed periodically since some patients who go on to develop an ATD could have specific anomalies on ultrasound prior to any other anomaly. Ultrasound scans are strongly recommended in the presence of altered TSH levels or positivity of thyroid autoimmunity markers. In cases of subclinical thyroid disease, thyroid function and autoimmunity should be assessed more frequently. Therapy should be appropriate and administered as soon as possible after a diagnosis of overt ATD is made.

## Figures and Tables

**Table 1:** Major nonimmunologic clinical features in patients with 22q11.2DS.

Clinical Features	All 22q11.2DS Patients
**Hypocalcemia (mild and severe)**	53/73 (72%)
**Cardiac anomalies**	46/73 (63%)
**Combined cardiac defect (e.g., Fallot)**	25/73 (34%)
**Isolated cardiac defect (e.g., IVD)**	21/73 (29%)
**Developmental delay**	11/73 (15%)
**Neuropsychiatric disorders**	10/73 (13.7%)
**Failure to thrive**	8/73 (11%)
**Feeding difficulties due to palatal defect**	7/73 (9.5%)

**Table 2:** General features in patients with 22q11DS and ATD.

22q11.2DS and ATD	All	Female	Male	F:M Ratio	Mean Age at Diagnosis (Years and DS)
**All ATD under 18**	16/73 (21.9%)	13	3	4.33	10.34 ± 2.87
**HT**	15/73 (20.5%)	13	2	6	10.28 ± 2.96
**GD**	1/73 (1.4%)	0	1	-	11.29

**Table 3:** Thyroid Morphological Ultrasound Features in 22q11DS patients.

Morphologic Feature on US	22q11.2DS ATD+	22q11.2DS ATD-	*p*-Value	RR Value	95% CI
Overflow	9/16	1/42	<0.0001	6.171	3.031 to 12.67
Nodules/pseudonodules	3/16	4/42	0.3813	1.681	0.5714 to 3.744
Inhomogeneity	13/16	14/42	0.014	4.975	1.752 to 15.18
Hypoecogenity	10/16	8/42	0.032	3.704	1.624 to 8.531
Perithyroidal lymphoadenopathy	4/16	2/42	0.043	2.889	1.163 to 5.568
Structural anomalies	7/16	7/42	0.0096	3.278	1.433 to 6.986

**Table 4:** Lymphocyte Subset Analysis in 22q11DS with and without ATD.

Lymphocyte Subset	22q11.2DS ATD+ (Mean and DS)	22q11.2DS ATD− (Mean and DS)	*T*-Student (*p* < 0.05)
CD3+	982.88 ± 319.1	1044.52 ± 398.79	*p* = 0.287244
CD3+CD4+	537 ± 213.8	603.65 ± 237.03	*p* = 0.159249
CD3+CD8+	329.25 ± 63.46	341.46 ± 171.24	*p* = 0.396863
CD19+	312.13 ± 152.14	461.38 ± 305.28	*p* = 0.03239
CD4+CD45RA+ %	46.56 ± 15.51	47.72 ± 18.59	*p* = 0.412255
CD4+CD45RO+ %	52.19 ± 16.17	51.80 ± 12.36	*p* = 0.471045
CD4+CD45RA+CD31+ %	81.43 ± 15.21	77.59 ± 12.36	*p* = 0.174093
CD27+IgM+IgD− %	8.16 ± 4.94	7.62 ± 9.45	*p* = 0.399932

**Table 5:** ATD prevalence in our 22q11DS cohort and literature and in Italian general population.

	22q11.2DS Cohort Prevalence	Shugar et al. [14]. Prevalence	Pediatric Italian General Population Prevalence [28]	z-Score for One Proportion (95% CI)
All ATD	21.9%	9.5%	1.2%	***p* < 0.00001**

Z-score values for one proportion (95%CI) between the prevalence described in this study and the one reported in the general population.

## Data Availability

For reasons of privacy and confidentiality, the data from this study are available from the corresponding authors upon reasonable request.

## References

[1] Kyritsi E.M., Kanaka-Gantenbein C. (2020). Autoimmune Thyroid Disease in Specific Genetic Syndromes in Childhood and Adolescence. Front. Endocrinol..

[2] Goodship J., Cross I., Liling J., Wren C. (1998). A population study of chromosome 22q11 deletions in infancy. Arch. Dis. Child..

[3] Roby B.B., Broderick M., Bohm L.A., Lesperance M. (2021). 22q11.2 Deletion Syndrome. Cummings Pediatric Otolaryngology.

[4] McLean-Tooke A., Spickett G.P., Gennery A.R. (2007). Immunodeficiency and autoimmunity in 22q11.2 deletion syndrome. Scand. J. Immunol..

[5] Jerome L.A., Papaioannou V.E. (2001). DiGeorge syndrome phenotype in mice mutant for the T-box gene, Tbx1. Nat. Genet..

[6] de Almeida J.R., James A.L., Papsin B.C., Weksburg R., Clark H., Blaser S. (2009). Thyroid gland and carotid artery anomalies in 22q11.2 deletion syndromes. Laryngoscope.

[7] Stagi S., Lapi E., Gambineri E., Salti R., Genuardi M., Colarusso G., Conti C., Jenuso R., Chiarelli F., Azzari C. (2010). Thyroid function and morphology in subjects with microdeletion of chromosome 22q11 (del(22)(q11)). Clin. Endocrinol..

[8] Jawad A.F., McDonald-McGinn D.M., Zackai E., Sullivan K.E. (2001). Immunologic features of chromosome 22q11.2 deletion syndrome (DiGeorge syndrome/velocardiofacial syndrome). J. Pediatr..

[9] Simmonds M.J., Gough S.C.L. (2005). Genetic insights into disease mechanisms of autoimmunity. Br. Med. Bull..

[10] Dejaco C., Duftner C., Grubeck-Loebenstein B., Schirmer M. (2006). Imbalance of regulatory T cells in human autoimmune diseases. Immunology.

[11] Simmonds M.J., Gough S.C.L. (2004). Unravelling the genetic complexity of autoimmune thyroid disease: HLA, CTLA-4 and beyond. Clin. Exp. Immunol..

[12] Dayan C.M., Daniels G.H. (1996). Chronic autoimmune thyroiditis. N. Engl. J. Med..

[13] Dittmar M., Libich C., Brenzel T., Kahaly G.J. (2011). Increased familial clustering of autoimmune thyroid diseases. Horm. Metab. Res..

[14] Shugar A.L., Shapiro J.M., Cytrynbaum C., Hedges S., Weksberg R., Fishman L. (2015). An increased prevalence of thyroid disease in children with 22q11.2 deletion syndrome. Am. J. Med. Genet. A.

[15] Caturegli P., de Remigis A., Rose N.R. (2014). Hashimoto thyroiditis: Clinical and diagnostic criteria. Autoimmun. Rev..

[16] Menconi F., Marcocci C., Marinò M. (2014). Diagnosis and classification of Graves’ disease. Autoimmun. Rev..

[17] Ueda D. (1989). Sonographic measurement of the volume of the thyroid gland in healthy children. Acta Paediatr. Jpn..

[18] Ohta T., Nishioka M., Nakata N., Fukuda K., Shirakawa T. (2018). Significance of Perithyroidal Lymph Nodes in Benign Thyroid Diseases. J. Med. Ultrason..

[19] Hakim F.T., Gress R.E. (2005). Reconstitution of the lymphocyte compartment after lymphocyte depletion: A key issue in clinical immunology. Eur. J. Immunol..

[20] Brent G.A. (2010). Environmental exposures and autoimmune thyroid disease. Thyroid.

[21] Brown R.S. (2013). Autoimmune thyroiditis in childhood. J. Clin. Res. Pediatr. Endocrinol..

[22] Morshed S.A., Latif R., Davies T.F. (2012). Delineating the autoimmune mechanisms in Graves’ disease. Immunol. Res..

[23] Zaletel K., Gaberšček S. (2011). Hashimoto’s Thyroiditis: From Genes to the Disease. Curr. Genom..

[24] Cappa M., Bizzarri C., Crea F. (2010). Autoimmune thyroid diseases in children. J. Thyroid Res..

[25] Driscoll D.A., Spinner N.B., Budarf M.L., McDonald-McGinn D.M., Zackai E.H., Goldberg R.B., Shprintzen R.J., Saal H.M., Zonana J., Jones M.C. (1992). Deletions and microdeletions of 22q11.2 in velo-cardio-facial syndrome. Am. J. Med. Genet..

[26] Wilson D.I., Burn J., Scambler P., Goodship J. (1993). DiGeorge syndrome: Part of CATCH 22. J. Med. Genet..

[27] Ryan A.K., Goodship J.A., Wilson D.I., Philip N., Levy A., Seidel H., Schuffenhauer S., Oechsler H., Belohradsky B., Prieur M. (1997). Spectrum of clinical features associated with interstitial chromosome 22q11 deletions: A European collaborative study. J. Med. Genet..

[28] Stagi S., Giani T., Simonini G., Falcini F. (2005). Thyroid function, autoimmune thyroiditis and coeliac disease in juvenile idiopathic arthritis. Rheumatology.

[29] Gleeson P.A., Toh B.H., van Driel I.R. (1996). Organ-specific autoimmunity induced by lymphopenia. Immunol. Rev..

[30] Khoruts A., Fraser J.M. (2005). A causal link between lymphopenia and autoimmunity. Immunol. Lett..

[31] Min B., McHugh R., Sempowski G.D., Mackall C., Foucras G., Paul W.E. (2003). Neonates support lymphopenia-induced proliferation. Immunity.

[32] Mackall C.L., Hakim F.T., Gress R.E. (1997). Restoration of T-cell homeostasis after T-cell depletion. Semin. Immunol..

[33] Ricci S., Masini M., Valleriani C., Casini A., Cortimiglia M., Grisotto L., Canessa C., Indolfi G., Lippi F., Azzari C. (2018). Reduced frequency of peripheral CD4+CD45RA+CD31+ cells and autoimmunity phenomena in patients affected by Del22q11 syndrome. Clin. Immunol..

[34] Tison B.E., Nicholas S.K., Abramson S.L., Hanson I.C., Paul M.E., Seeborg F.O., Shearer W., Perez M.D., Noroski L.M., Chinen J. (2011). Autoimmunity in a cohort of 130 pediatric patients with partial DiGeorge syndrome. J. Allergy Clin. Immunol..

[35] Montin D., Marolda A., Licciardi F., Robasto F., Di Cesare S., Ricotti E., Ferro F., Scaioli G., Giancotta C., Amodio D. (2019). Immunophenotype Anomalies Predict the Development of Autoimmune Cytopenia in 22q11.2 Deletion Syndrome. J. Allergy Clin. Immunol. Pract..

[36] Stożek K., Grubczak K., Marolda V., Eljaszewicz A., Moniuszko M., Bossowski A. (2020). Lower proportion of CD19^+^IL-10^+^ and CD19^+^CD24^+^CD27^+^ but not CD1d^+^CD5^+^CD19^+^CD24^+^CD27^+^ IL-10^+^ B cells in children with autoimmune thyroid diseases. Autoimmunity.

[37] Frommer L., Kahaly G.J. (2021). Type 1 Diabetes and Autoimmune Thyroid Disease—The Genetic Link. Front. Endocrinol..

